# Coexistence of a colon carcinoma with two distinct renal cell carcinomas: a case report

**DOI:** 10.1186/1752-1947-5-134

**Published:** 2011-04-04

**Authors:** Alexandros E Papalampros, Athanasios S Petrou, Eleftherios I Mantonakis, Konstantinos I Evangelou, Lambros A Giannopoulos, Georgios G Marinos, Athanasios L Giannopoulos

**Affiliations:** 1 First Department of Surgery, University of Athens, Laiko General Hospital, Greece; 2 Molecular Carcinogenesis Group, Department of Histology and Embryology, Medical School, University of Athens, Greece

## Abstract

**Introduction:**

We present the case of a patient with two tumors in his left kidney and a synchronous colon cancer. While coexisting tumors have been previously described in the same kidney or the kidney and other organs, or the colon and other organs, to the best of our knowledge no such concurrency of three primary tumors has been reported in the literature to date.

**Case presentation:**

A 72-year-old man of Greek nationality presenting with pain in the right hypochondrium underwent a series of examinations that revealed gallstones, a tumor in the hepatic flexure of the colon and an additional tumor in the upper pole of the left kidney. He was subjected to a right hemicolectomy, left nephrectomy and cholecystectomy, and his postoperative course was uneventful. Histopathology examinations showed a mucinous colon adenocarcinoma, plus two tumors in the left kidney, a papillary renal cell carcinoma and a chromophobe renal cell carcinoma.

**Conclusion:**

This case underlines the need to routinely scan patients pre-operatively in order to exclude coexisting tumors, especially asymptomatic renal tumors in patients with colorectal cancer, and additionally to screen concurrent tumors genetically in order to detect putative common genetic alterations.

## Introduction

Synchronous multiple primary tumors are relatively rare. The etiology and pathogenesis of such multiple tumors remain unclear. It has been hypothesized that concurrent tumors can arise from tissues with similar embryological origin when they are simultaneously affected by factors such as carcinogens or hormones. Coexisting tumors in the colon and kidney are more often diagnosed nowadays due to the widespread use of ultrasonography and computed tomography (CT) or magnetic resonance imaging (MRI) techniques.

### Case presentation

**Figure 1:**
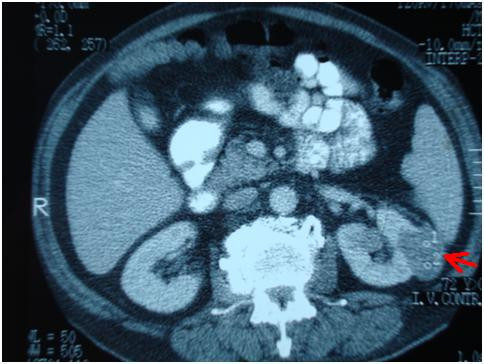
**Computed tomography (CT) scan showing the tumor at the external margin of the left kidney (red arrow)**.

**Figure 2:**
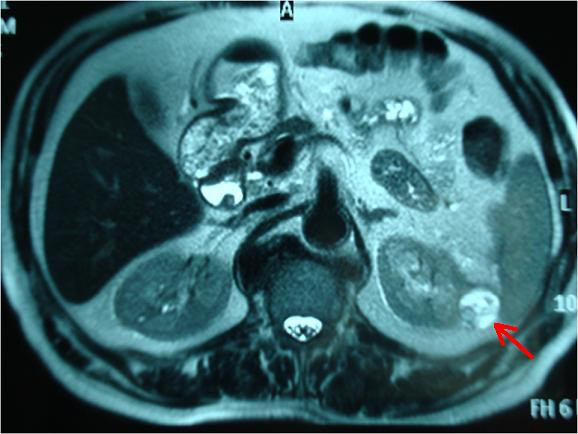
**MRI scan showing the tumor at the external margin of the left kidney (red arrow)**.

**Figure 3:**
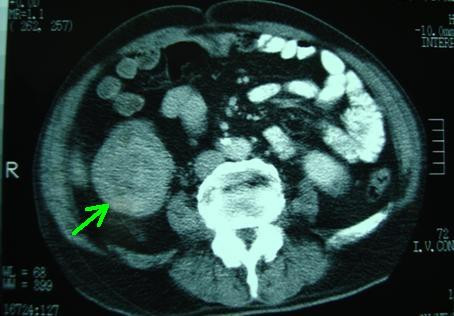
**Computed tomography (CT) scan showing distension of the ascending colon with concomitant wall thickening and dimness of the pericolic fat tissue (green arrow)**.

A 72-year-old man of Greek nationality presented to our facility with pain in the right hypochondrium. He underwent an abdominal ultrasound, which revealed multiple gallstones and a 4 × 3.3 cm tumor in the upper pole of the left kidney. Abdominal CT and MRI scans showed a 4 cm solid tumor at the external margin of the left kidney that extended up to the neighboring surface of the spleen (red arrows in Figures 1 and 2). The scans also showed a distension of the ascending colon with concomitant wall thickening and dimness of the pericolic fat tissue (green arrow in Figure 3), findings indicating possible neoplasia.

Our patient was then admitted to our clinic for further examination and treatment. From his medical history he was a smoker of 50 packs/year, had arterial hypertension and had reported alternating diarrhea and constipation during the last five years, with no family history as far as malignancies were concerned. A physical examination revealed a palpable mass in the right subcostal region. Laboratory data on admission revealed hypochromic anemia, with a hemoglobin level of 11.1 g/dL and an α-fetoprotein level of 7.61 ng/dL; all other tumor markers were found to be at normal levels. Colonoscopy revealed a mass in the hepatic flexure, while a dimercaptosuccinic acid scan showed that both kidneys were functioning normally.

Our patient underwent surgery and was subjected to a right hemicolectomy, left nephrectomy and cholecystectomy. Reconstruction was performed by an end-to-side ileo-transversostomy. He had an uneventful postoperative course and was discharged nine days later.

The following two specimens were obtained by our department of pathology for histopathological examination:

 (1) right hemicolectomy composed of a portion of terminal ileum, cecum with the appendix and ascending colon with the corresponding pericolic fat tissue. Grossly, an exophytic grayish tumor (size 5.5 × 5 × 5 cm) was detected in the cecum near the ascending colon area. Macroscopically the tumor seemed to extend through the colonic wall. After processing, 27 lymph nodes were found in the pericolic fat.

(2) Left nephrectomy composed of left kidney (size 12 × 9 × 4 cm), ureter stump and perinephric tissue. Grossly, two tumors were recognized: one occupied the upper pole of the kidney (size 4.5 × 4 cm), was whitish and friable, and was restricted under the fibrous capsule; the second (size 2.2 × 1.9 cm) was a gray-brown, well circumscribed, solitary mass, with regions of hemorrhage and necrosis, near the lower pole. The distance between these lesions was approximately 5.5 cm.

### Microscopic features

**Figure 4:**
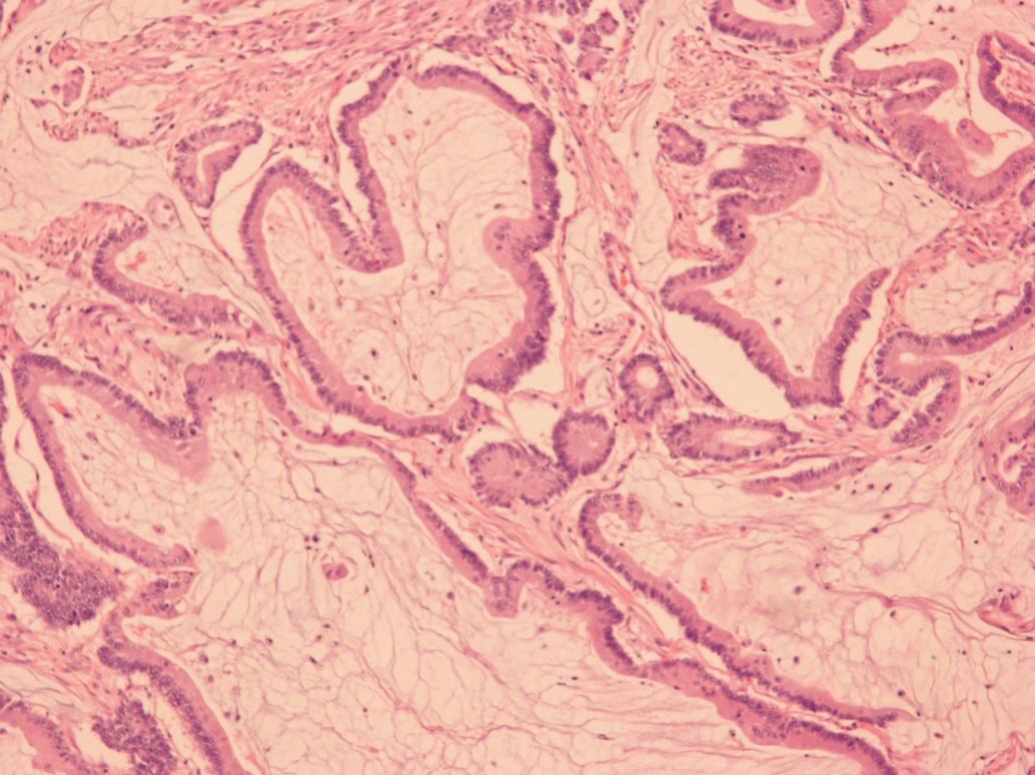
**Representative area of the mucinous colon adenocarcinoma, depicting malignant epithelium within pools of extracellular mucin (hematoxylin and eosin counterstain, magnification ×100)**.

At the histological level we observed pools of extracellular mucin (>50% of the neoplastic tissue was composed of mucin) that contained single cancer cells and a malignant epithelium that formed acinar structures and/or cellular strips. The carcinoma penetrated through the muscularis propria of the bowel wall. No lymph node metastasis was detected. The diagnosis made was mucinous colon adenocarcinoma, stage Dukes B (Figure 4).

**Figure 5:**
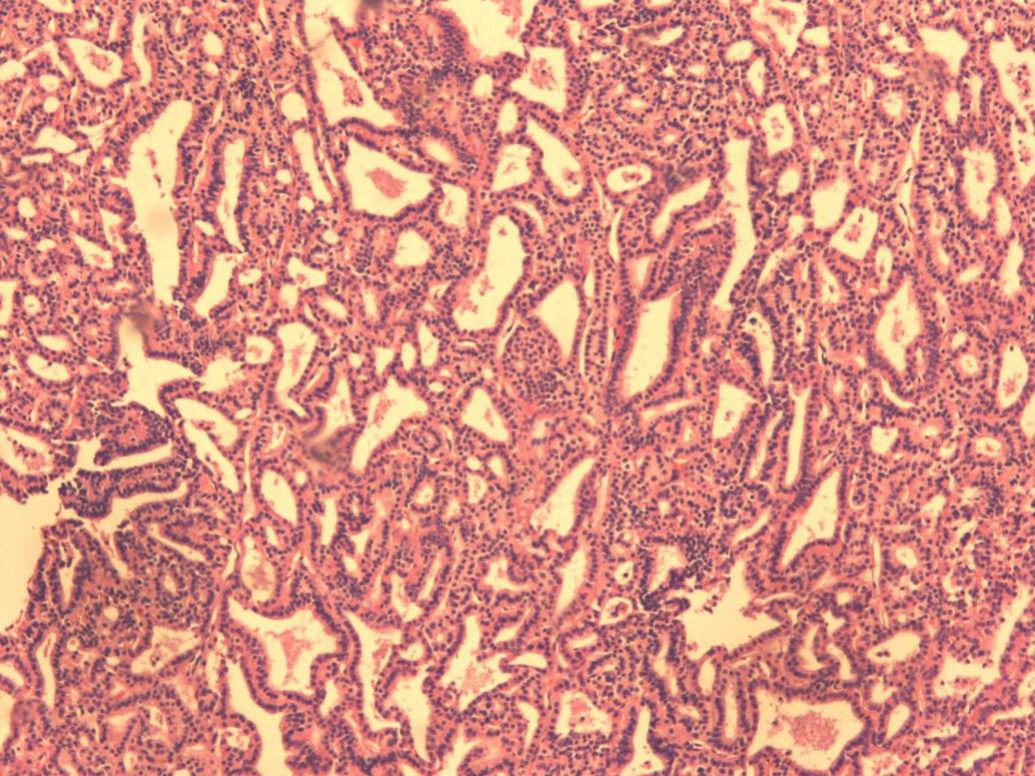
**Histological section of the papillary renal cell carcinoma of the upper pole with a papillary, tubulopapillary and cystic growth pattern of cancer cells (hematoxylin and eosin counterstain, magnification ×100)**.

**Figure 6:**
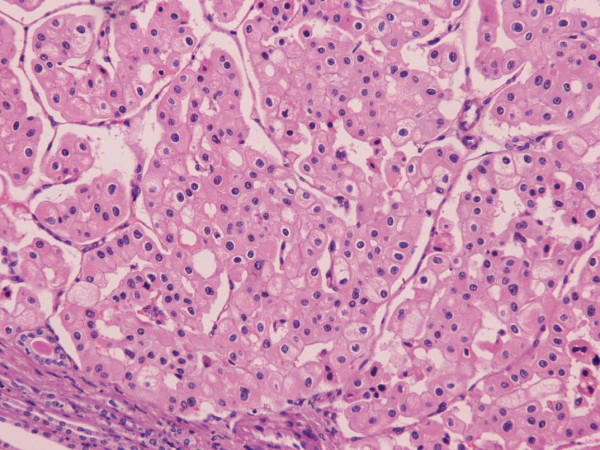
**Microscopic view of the chromophobe renal cell carcinoma of the lower pole**. Large round cells with abundant cytoplasm, a clear perinuclear halo and hyperchromatic nuclei are evident (hematoxylin and eosin counterstain, magnification ×200).

Histological analysis of the renal tumor in the upper pole revealed a carcinoma, with papillary tubulopapillary and cystic growth pattern accompanied by fibrovascular cores and aggregates of foamy macrophages (Figure 5). The papillae were mostly lined by neoplastic cells with high nuclear grade, eosinophilic cytoplasm and pseudostratified nuclei and focally by small cells with scanty cytoplasm arranged in a single layer. Immunohistochemically, the tumor cells exhibited strong cytokeratin 7 immunoreactivity. The second tumor corresponded to a carcinoma with a solid growth pattern. Large round cells with abundant cytoplasm, a clear perinuclear halo and hyperchromatic nuclei were observed. Binucleated and multi-nucleated tumor cells were also present. The tumor cells were epithelial membrane antigen (EMA) and cytokeratin immunopositive, and vimentin negative, while Hale's colloidal iron staining showed a reticular and diffuse staining pattern (Figure 6). Histopathological analysis led to a diagnosis of papillary renal cell carcinoma type 2 and focally type 1, grade 2 to 3; Furhman grading system, (pT1b) for the tumor of the upper pole (Figure 5) and chromophobe renal cell carcinoma (pT1a) for the tumor of the lower pole of the kidney (Figure 6), respectively.

## Discussion

In our patient, we observed the coexistence of three individual primary tumors, one in the colon and two in the left kidney. These tumors covered the criteria set by Warren and Gates for the diagnosis of multiple primary malignant synchronous tumors [1]. To the best of our knowledge, no case with such a concurrency has been reported to date in the literature (Table 1 and [2-21]).

**Table 1:** Cases of primary renal tumors coexisting with other primary malignancies

Renal tumor(s)	Synchronous tumor(s)	Reference
Renal cell carcinoma	Colorectal carcinoma	Halak *et al. *[2]

Renal cell carcinoma	Gynecological malignancies	Cheung Wong *et al. *[3]

Chromophobe cell carcinoma	Papillary renal carcinoma	Tyritzis *et al. *[4]

Renal cell carcinoma	Other sites	Beisland *et al. *[5]

Renal cell carcinoma	Esophageal carcinoma	Kobayashi *et al. *[6]

Papillary renal carcinoma	Heart liposarcoma	Gałazka *et al. *[7]

Renal oncocytoma	Endometrioid ovarian and endometrial carcinoma	Bezircioğlu *et al. *[8]

Chromophobe cell carcinoma	Urothelial carcinoma	Joon Choi *et al. *[9]

Ipsilateral renal cell carcinoma	Urothelial carcinoma of the renal pelvis	Leveridge *et al. *[10]

Renal cell carcinoma	Perirenal liposarcoma	Kinebuchi *et al. *[11]

Renal cell carcinoma	Central nervous system lymphoma	Chang *et al. *[12]

Renal cell carcinoma	Extragonadal retroperitoneal teratoma	Ambani *et al. *[13]

Renal cell carcinoma	Non-Hodgkin's lymphoma (T cell type)	Khadilkar *et al. *[14]

Chromophobe cell carcinoma	Carcinoid tumor of the gallbladder	Morelli *et al. *[15]

Renal cell carcinoma	Papillary renal carcinoma/chromophobe cell carcinoma	Petrolla *et al. *[16]

Renal cell carcinoma	Malignant lymphoma	Yagisawa *et al. *[17]

Transitional cell carcinoma	Right colon cancer	Kumar *et al. *[18]

Malignant rhabdoid tumor	Brain tumor	Adachi *et al. *[19]

Renal cell carcinoma	Uterine cervical adenocarcinoma	Yokoyama *et al. *[20]

Cystic renal cell and squamous cell carcinoma	Transitional cell carcinoma of ipsilateral ureter and urinary bladder	Charles *et al. *[21]

An exophytic grayish tumor (size 5.5 × 5 × 5 cm) was localized in the cecum and diagnosed as a mucinous colon adenocarcinoma with no lymph node metastasis; stage Dukes B. These tumors include many high-frequency microsatellite instability (MSI-H) carcinomas. It has been suggested that the MSI status influences the aggressiveness of this histopathological subtype [22]. When these tumors develop in the rectum they exhibit the poorest overall prognosis [23]. However, other studies report that no significant difference in prognosis between mucinous and non-mucinous types of adenocarcinoma exists.

In the left kidney two tumors were observed. The first one, whitish and friable (size 4.5 × 4 cm), occupying the upper pole of the kidney and the other (size 2.2 × 1.9 cm) presenting as a gray-brown well circumscribed, solitary mass located near the lower pole. Histopathological examination revealed papillary renal cell carcinoma type 2 and focally type 1, grade 2 to 3 (pT1b) for the first tumor and chromophobe renal cell carcinoma (pT1a) for the second.

Papillary renal cell carcinomas (PRCCs) comprise approximately 10% of renal cell carcinomas and are known to originate from the distal convoluted tubule. The most common genetic aberrations detected in these carcinomas are trisomy or tetrasomy 7, trisomy 17 and loss of chromosome Y. Other alterations reported are interstitial loss of heterozygosity (LOH) at 3p, trisomy 12, 16 and 20 related to tumor progression and LOH at 9p13 that is associated with shorter survival. In other studies comparative genome hybridization (CGH) analysis revealed more gains of chromosomes 7p and 17p in type 1 carcinomas in comparison to type 2 tumors, while different types of allelic imbalance at 17q and 9p have also been described. PRCC seems overall to have a better prognosis than clear cell carcinomas of the same stage and grade and at lower stages and grades, but the prognosis is about the same for higher stages and grades [24]. The five-year survival has been reported to range from 49% to 84% [25]. Type I seems to have a significantly better prognosis than clear cell carcinoma while type II has about the same prognosis as clear cell carcinoma. Factors such as tumor grade, stage and sarcomatoid dedifferentiation influence the patient's outcome.

Chromophobe renal cell carcinoma is a relatively rare malignancy that comprises 5% of renal cell carcinomas [24] and is described to arise from the intercalated cells of the distal convoluted tubule. These tumors exhibit good prognosis with a mortality rate of less than 10% [26]. The main genetic aberrations that characterize these carcinomas are losses of chromosomes 1, 2, 6, 10, 13, 17 and 21, hypodiploid DNA context, as well as telomere shortening. P53 mutations in 27% of cases and LOH at the 10q23.3 chromosomal region have also been reported.

Many cases of histological distinct renal tumors occurring coincidentally in the same patients have been reported [27]. Some of them describe coexistence of renal cell carcinoma with a benign tumor, such as oncocytoma, angiomyolipoma, leiomyoma and adrenal adenoma. Others refer to familial cancer syndromes, which consist of multiple cancers in a single patient or the presentation of cancer at an earlier age or more than one family members with the same cancer. For example, urothelial cancer has been associated with Lynch syndrome. Two or three concurrent renal cell tumors have been reported in cases of hybrid tumors [28] and in Birt-Hogg-Dubé syndrome, but also in sporadic cases [16]. A recent report by Tyritzis *et al. *describes the case of a 57-year-old man with synchronous chromophobe and papillary renal cell carcinoma within the same kidney. The authors assumed that different renal tumors could arise from cancer stem cells that follow dissimilar differentiation pathways regulated by tissue microenvironmental interactions [29]. Another hypothesis was the evolution of one subtype to another (as oncocytomas, for example, posses the ability to evolve into papillary carcinomas [30]), or that one malignant renal tumor could switch to another type.

The simultaneous occurrence of renal cell carcinoma with malignancies that develop in other sites has also been documented. Such malignancies include urological cancers [10], esophageal carcinomas, colorectal carcinomas [2], lung cancer, breast cancer, gynecological cancer, sarcoma and non-Hodgkin's lymphoma. It has been estimated that urogenital and gastrointestinal tumors were the most common pairing of synchronous cancers [1]. However, the pathogenetic mechanism responsible remains unknown.

In a recent study it has been shown that patients with urological cancer (cancer of the ureter or renal pelvis, and to a lesser extent patients with bladder or renal parenchymal cancer) were found to be at a higher risk for developing subsequent colon carcinoma than the general population and vice versa. The authors assumed that this two-directional association might be driven by common environmental risk factors (smoking, diet, carcinogens), screening bias, a shared genetic predisposition (mismatch repair defect) or by the effect of treatment of one type of cancer on the other. As far as genetic predisposition is concerned, it is well established that in hereditary non-polyposis colon cancer (HNPCC) various mismatch repair genes are functionally affected. Individuals with a mutation in one of these genes have an 80% lifetime risk of developing colon cancer [31] and a well established increased risk of developing extracolonic tumors, including endometrial, ovarian, ureteral, and renal cancers [32]. Additionally, microsatellite instability testing in patients with HNPCC has been described as a cost-effective and feasible method for identifying candidates for HNPCC testing, indicating that microsatellite instability testing could be applied to patients with colorectal and urological cancers.

## Conclusion

This report describes for the first time the coexistence of a colon carcinoma with a combination of two distinct renal cell carcinomas with different histological subtypes, papillary and chromophobe, within the left kidney. Such cases underline the need to perform routine pre-operative imaging studies to exclude synchronous asymptomatic renal tumors in patients with colorectal cancer, and after surgery to genetically analyze synchronous tumors in view of detecting common genetic aberrations.

## Consent

Written informed consent was obtained from the patient for publication of this case report and any accompanying images. A copy of the written consent is available for review by the Editor-in-Chief of this journal.

## Competing interests

The authors declare that they have no competing interests.

## Authors' contributions

AP, AP, GM and AG were involved in acquiring our patient's history, examinations, participated in his treatment (surgery, hospitalization, and so on) and in the acquisition and interpretation of data. EM and LG participated in writing and revising the manuscript. KE participated in examining the histopathology specimens, reviewing the literature and submitting his report (included in the Discussion) to us. AG was also responsible for the final approval and supervision of the manuscript. All authors read and approved the final manuscript.
